# An Approach to Histopathological Audit in Bristol

**Published:** 1986-04

**Authors:** J. H. F. Smith, S. Z. Al-Sam, J. C. Briggs, J. D. Davies

**Affiliations:** University Department of Pathology, Bristol Royal Infirmary and the Department of Histopathology, Frenchay Hospital; University Department of Pathology, Bristol Royal Infirmary and the Department of Histopathology, Frenchay Hospital; University Department of Pathology, Bristol Royal Infirmary and the Department of Histopathology, Frenchay Hospital; University Department of Pathology, Bristol Royal Infirmary and the Department of Histopathology, Frenchay Hospital

## Abstract

There are many possible methods of histopathological audit. Most are expensive of both time and finance. We present an outline of one which is relatively cheap and is based on a meeting that has taken place in Bristol for many years. An analysis of the material reviewed in one year at these meetings indicates that they encompass a wide range of pathology. Furthermore it is possible to get some quantitative indication of the type of lesions that most frequently pose diagnostic problems, and which are thought to be potentially soluble by the opinion of other general histopathologists. The contribution of this type of meeting to histopathological audit is considered.


### An approach to histopathological audit in Bristol

**J. H. F. Smith, S. Z. Al-Sam, J. C. Briggs and J. D. Davies***
University Department of Pathology, Bristol Royal Infirmary and the Department of Histopathology, Frenchay Hospital.

### SUMMARY

There are many possible methods of histopathological audit. Most are expensive of both time and finance. We present an outline of one which is relatively cheap and is based on a meeting that has taken place in Bristol for many years. An analysis of the material reviewed in one year at these meetings indicates that they encompass a wide range of pathology. Furthermore it is possible to get some quantitative indication of the type of lesions that most frequently pose diagnostic problems, and which are thought to be potentially soluble by the opinion of other general histopathologists. The contribution of this type of meeting to histopathological audit is considered.

### INTRODUCTION

In recent years the subject of clinical audit has arisen in several fields. Involvement of pathologists has been advocated in some of these exercises. However pathologists themselves also require similar appraisal of their own laboratory performances. National External Quality Assessment schemes (Whitehead and Woodford, 1981) are in fact already operating in clinical pathology disciplines, and in the technical aspects of histopathology (Barr and Williams, 1982). Such nationally based assessments have not been considered feasible in diagnostic histopathology, although a local scheme has been described in the South Western Region involving pathologists from Exeter, Torbay, Plymouth and Truro (Sherwood and Hunt, 1984) in retrospective diagnosis of circulated sections.

We wish to describe another method of histopathological audit which takes place in Bristol. This too is locally based, but is mainly concerned with current cases rather than in reviewing old material. For many years a weekly meeting for the diagnostic histopathologists in Bristol and surrounding areas has taken place for one hour on Friday mornings. In Spring 1984 it was agreed that a survey of the group's activity should be conducted. We report the first year's findings of the survey, and a preliminary audit of the areas of difficulty in histological diagnosis.

### MATERIALS AND METHOD

A weekly meeting for diagnostic histopathologists in Bristol currently takes place in the Lecture Theatre of the Bristol Children's Hospital where a powerful Nikon projection microscope, 2×2 transparency, and overhead projectors are available. These facilities allow appraisal of both low- and high-power magnifications of the tissues that are discussed.

*Address for correspondence: Dr. J. D. Davies, University Department of Pathology, Bristol Royal Infirmary, Bristol BS2 8HW.

The meetings, which are entirely voluntary, have involved 11 Consultants, 9 Senior Registrars, 4 Registrars, 4 Senior House Officers (SHOs) and 2 Clinical Assistants. The 30 pathologists came from the Royal United Hospital, Bath (2), the General Hospital, Weston-super-Mare (1), the Royal Devon and Exeter Hospital (1), Frenchay Hospital (5), Southmead Hospital (6), Bristol Children's Hospital (2), Bristol General Hospital (1), and the Bristol Royal Infirmary (12). In addition four veterinary histopathologists from the School of Veterinary Science, Langford have also taken an active part in the proceedings. The meeting is usually chaired by a senior University (JDD) or National Health Service (JCB) consultant histopathologist. During the meeting cases are presented which have either general diagnostic or pathogenetic interest, or, which are submitted for diagnostic opinion. Any individual case may fall into more than one of the foregoing categories.

In the last year, with the consent of the participants in the group, a record has been kept (JHFS and SZAS) of the cases discussed, the presenting pathologist's diagnosis, and of the eventual diagnostic consensus that was reached by the meeting.

### RESULTS

In the one-year period 18 May 1984 to 10 May 1985 40 meetings were surveyed. In those meetings 253 human histopathological cases were discussed by the participants. Consultants presented 132 of the cases (52.2% of the total), senior registrars presented 94 cases (37.1%), registrars presented 21 cases (8.3%), an SHO presented 1 case (0.4%) and the clinical assistants presented 5 cases (2.0%). An average of 6.3 cases was considered at each meeting. In addition, the four veterinary pathologists presented 16 cases of various spontaneous animal lesions (these are not included in the analysis below).

Of the human cases a total of 253 was shown from 37 different anatomical sites. The vast majority of the cases were biopsies or surgical excisions; only few were postmortem demonstrations. Skin (48 cases), soft tissue including peripheral-nerve lesions (27), lung (16), large bowel (15), lymph node (14), brain (12), breast and kidney (11 each) and 9 foetal or neonatal cases accounted for the majority of the material presented. Fewer lesions were shown from liver (8 cases), small bowel (8), stomach (7), and five each of ENT tissue, salivary glands, and meninges. Four cases from ovary, heart, appendix, mediastinum and uterus, and, three of spleen, bone, spinal cord, muscle, adrenal and oesophagus were discussed. Two cases involving tongue, testis and vulva, and one each of joint and synovium, pleura, gall bladder, vagina, carotid body, prostate, pituitary and oral cavity accounted for the remainder.

Of those presented the majority (191; 75.5%) were cases in which a confident diagnosis had already been reached. The reason for showing such cases was usually that the lesion showed some peculiar features or possessed diagnostic lessons. In others there were atypical appearances, or else the case was an example of a classical but rare entity. However in 62 of the 253 cases (24.5%) there was genuine doubt as the diagnosis on the part of the presenting pathologist, and the case was brought for the opinion of the meeting. Breast lesions (7 of 11; 64%), soft-tissue tumours (13 of 27; 48%), lung lesions (7 of 16; 44%), lymph nodes (6 of 14; 43%) and liver biopsies (3 of 7; 43%) accounted for the majority of this diagnostically difficult group (Table 1). Only 7 of the 48 skin lesions (15%) were presented as diagnostic problems. Of the remaining cases presented in this way two each were derived from brain, large bowel, muscle biopsies and small bowel, and a single case from bone, mediastinum, uterus, adrenal, heart, stomach, testis, oral cavity, salivary gland and vagina.

**Table 1**

**Tissue of origin and proportions of cases brought for diagnostic opinion at histopathology meeting**

| *Organ* | *No. cases discussed* | *Cases brought for opinion No.* | *Percentage that organ* |
|---|---|---|---|
| Breast | 11 | 7 | 64% |
| Soft tissue | 27 | 13 | 48% |
| Lung | 16 | 7 | 44% |
| Lymph nodes | 14 | 6 | 43% |
| GI & liver | 45 | 8 | 18% |
| Miscellaneous | 69 | 12 | 17% |
| Brain | 12 | 2 | 16% |
| Skin | 48 | 7 | 15% |
| Kidney | 11 | 0 | 0% |
| Total | 253 | 62 | 25% |

All 62 cases presented for diagnostic opinion by the group were given a provisional diagnosis by the pathologist who demonstrated the case to the meeting. In 40 cases both the original and the consensus diagnosis reached at the meeting was the same. In 22 cases (35.5%) the two diagnoses differed. These discrepancies were found in a wide range of organs (Table 2). However the lack of concordance of diagnoses represented 33% of lymph nodes, 31% of soft-tissue lesions, and 29% of breast and lung cases which were specifically brought for diagnostic opinion. In only one of the 7 skin lesions in this category (14.3%) was there a difference in the diagnoses (Table 2).

Most of the divergent diagnoses differed only in exact histogenetic typing of tumours (15 cases), or in the clinical significance of inflammatory reactions (2 cases). In two cases the consensus view was that artefactual difficulties precluded a precise diagnosis of muscular abnormalities (1 skeletal muscle; 1 colonic muscle). In only three cases was there disagreement about the benign or potentially malignant character of tumours or hyperplastic lesions.

Obviously the 253 cases presented at the meeting were highly selected, since the participants drew them from an estimated 57,000 surgical specimens received in their laboratories during the course of the period surveyed. The cases described therefore formed a mere 0.44% of the laboratory workloads. Those presented for diagnostic assistance were an even smaller proportion, being approximately only 1 case in 920, and the cases leading to divergent diagnoses were less than 1 in 2500.

### DISCUSSION

Few medical decisions are precise. Many necessarily involve subjective assessment and judgement. Histopathological diagnosis is no exception (King, 1967; Underwood, 1981; Davies, 1984), especially when the case is unusual or where several clinical, morphological and investigational factors are involved. Obviously there are simple diagnoses, but the type of case discussed in the meetings described tended to be complex or unusual.

There are several types of self-assessment schemes that are in use in histopathology. Most involve preselected cases which are circulated before discussion. This format is used for national and local Slide Clubs, departmental Black Box meetings, and in at least one External Quality Assessment Scheme (Sherwood and Hunt, 1984). Other uses of preselected slides are the educational testing of individual's diagnoses against those of 'experts' (Penner, 1973) or in diagnostic consistency studies (Ringsted *et al.*, 1978; Riddell *et al.*, 1983; MRC Breast Pathology Panel, 1985). Periodic clinical review sessions also usually employ this method. The type of meeting described in this paper offers a mixture of self-selected and preselected cases. Its virtues are the current nature of the cases, the ready availability of other opinions, and the opportunity for self-assessment. Within a city the size of Bristol, it is feasible to hold meetings much more frequently than is possible with most of the other methods listed above.

Clearly there are problems in any kind of audit. Unless matters are handled with tact such an exercise may present sensitive issues and unnecessarily provoke emotional fears. These attitudes seem best overcome by individual involvement with the group, thus allowing the potential benefits to be realised. The varied nature of the material, and its presentation by many members help to reduce such problems. Equally it is also beneficial if

**Table 2**

**Analysis of divergent initial and consensus diagnoses in various tissues**

| *Organ* | *No. divergent diagnoses* | *% Cases with divergent diagnoses* in: Cases for opinion* | *All cases discussed* |
|---|---|---|---|
| Breast | 2 | 29% | 18% |
| Soft tissue | 4 | 31% | 15% |
| Lymph nodes | 2 | 33% | 14% |
| Lung | 2 | 29% | 12% |
| Miscellaneous | 7 | 58% | 10% |
| GI & liver | 4 | 50% | 9% |
| Skin | 1 | 14% | 2% |
| Brain, kidney | 0 | 0% | 0% |
| Total | 22 | 35% | 9% |

*see Table 1 for denominators in various organs many members of the group are also able to express opinions in their own fields of particular interest. This spread of expertise is enhanced by the invitation of many histopathological visitors to Bristol to attend the meetings. These various approaches appear to reduce any authoritarian impression, and in themselves lead to more freedom in discussion.

Relatively small numbers of participants appear to have advantages in any histopathological audit. There has been little enthusiasm for large nationally based schemes. Indeed at a meeting held in May 1984 by the Department of Health and Social Security to discuss External Quality Assessment in Histopathology, the consensus opinion favoured small "cells" rather than national projects. Our experience confirms this view. The feed-back necessary for the success of such diagnostic exercises is certainly achievable with a group of up to 30 pathologists.

The range of organs discussed in the group meetings seems reasonably representative of the lesions involved in the more difficult types of histological interpretation. Naturally there are some excesses and some deficiences in the proportions of organs discusssed, but this probably results from individual enthusiasm, and from the very specialised nature of some branches of histopathology.

Finally as part of the auditing procedure, it is of interest to attempt to identify the areas of especial difficulty in diagnosis, as are brought to light by those cases which were presented especially for opinion, and by those in which the consensus view did not accord with the original proposed diagnosis. Lesions from the female breast, soft tissue, lymph nodes, lung and liver were in that order the most frequent causes of diagnostic concern—at least as was judged by the percentage of lesions that were specifically brought for opinion at the meeting (Table 1). A separate histopathological audit in Bristol of frozen-section diagnoses has already emphasised the relatively high proportion of errors in breast disease (Dankwa and Davies, 1985); it should however be made clear that the difficulties experienced with this organ are not just local nor are they restricted to frozen-section diagnosis (Kagali, 1983; MRC Breast Pathology Panel, 1985). In like manner difficulties are known to exist in soft-tissue tumours, lymph nodes, lung and liver biopsies. Surprisingly presentations for opinion were much less frequent in skin lesions, which is probably explained by the fact that nowadays such diagnostic difficulties are often better resolved by review meetings with the clinical dermatologists. For pathologists working with large clinical units similar considerations probably also apply in the fields of gastrointestinal, liver and renal biopsies. It is unlikely that many purely neuropathological or osseous lesions would benefit from appraisal by general histopathologists in a city which contains specialist pathology units for those tissues. None the less there are lesions in these anatomical sites which may represent disease processes that are systemic or arose primarily in other organs. The presentations of cases from these anatomical sites for general diagnostic opinion support this contention.

This method of histopathological audit appears to have several virtues. The obvious advantage of the approach is that it involves actual practical diagnosis rather than relying upon artificial retrospective appraisal. This is clearly closer to the day-to-day performance of the participants than can be any hurried, or even unusually detailed examination of formally circulated sets of histological sections. There are also two additional virtues in this type of audit. First it is educational for the trainees who take part, giving them insight into the processes of reaching a diagnostic conclusion. Second, it disseminates information about locally available diagnostic expertise and developments in new histopathological techniques. It is relatively flexible, is held at frequent intervals and is informal in style. The high proportion of local consultants and trainees who regularly attend indicate that it has some merit in their view. It is possible, as indicated above, to attempt to identify the areas which present especial diagnostic difficulties in current histopathological practice. It is hoped in future years to extend the analysis presented in this paper.

### ACKNOWLEDGEMENTS

We should like to acknowledge the encouragement of the late Dr. C. R. Tribe, who in fact originally suggested analysis of the case material. Our thanks are also due to all participants in the meeting, without the understanding and tact of whom the analysis would not have been possible. We also wish to acknowledge the secretarial support of Mrs. M. R. Lake and the financial help given by the Department of Health & Social Security to JDD for a pilot study of External Quality Assessment in Histopathology.

### REFERENCES

BARR, W. T. and WILLIAMS, E. D. (1982) Value of external quality assessment of the technical aspects of histopathology. *J.Clin.Pathol.* **35**, 1050–1056.

DANKWA, E. K. and DAVIES, J. D. (1985) Just how fallible are frozen-section diagnoses? *Bristol Med.-Chir.J.* **100**, 57.

DAVIES, J. D. (1984) In support of the colonoscopist: the pathologist *in* Gastrointestinal Endoscopy, ed. P. R. Salmon, Chapman & Hall, London, pp. 263–273.

KAGALI, V. A. (1983) The role and limitations of frozen section diagnosis of a palpable mass in the breast. *Surg.Gynecol.Obstet.* **156**, 168–170.

KING, L. S. (1967) How does a pathologist make a diagnosis? *Archs.Pathol.* **84**, 331–333.

MEDICAL RESEARCH COUNCIL BREAST PATHOLOGY PANEL (1985) Observer variability in histopathology reporting of breast lesions. *J. Clin. Pathol.* **38**, in press.

PENNER, D. W. (1973) Quality control and quality evaluation in histopathology and cytology. *Pathology Annual* **8**, 1–19.

RIDDELL, R. H., GOLDMAN, H., RANSOHOFF, D. F. *et al.* (1983) Dysplasia in inflammatory bowel disease: Standardized classification with provisional clinical applications. *Human Path.* **14**, 931–968.

RINGSTED, J., AMTRUP, F., ASKLUND, C. *et al.* (1978) Reliability of histopathological diagnosis of squamous epithelial changes of the uterine cervix. *Acta Pathol. Microbiol.Scand. Sect A* **86**, 273–278.

SHERWOOD, A. J., and HUNT, A. C. (1984) An external quality assessment scheme in histopathology. *J.Clin.Pathol.* **37**, 409–414.

UNDERWOOD, J. C. E. (1981) Introduction to biopsy interpretation and surgical pathology. Springer-Verlag, Berlin, p. 7 et seq.

WHITEHEAD, T. P. and WOODFORD, F. P. (1981) External quality assessment of clinical laboratories in the United Kingdom. *J.Clin.Pathol.* **34**, 947–957.

